# Role of LRRK2 in axonal transport and Parkinson’s disease

**DOI:** 10.1042/BCJ20253133

**Published:** 2025-06-25

**Authors:** Björn Twellsieck, C. Alexander Boecker

**Affiliations:** Department of Neurology, University Medical Center Goettingen, 37077 Goettingen, Germany

**Keywords:** axonal transport, LRRK2, RAB GTPases, Parkinson’s disease

## Abstract

Axonal transport is crucial for neuronal health and function, facilitating the delivery of newly synthesized material from the soma via anterograde transport and the removal of aged proteins and damaged organelles for degradation via retrograde transport. Emerging evidence links Parkinson’s disease (PD)-causing mutations in the leucine-rich repeat kinase 2 (*LRRK2*) gene to dysfunctional axonal transport. Pathogenic LRRK2 mutations induce increased LRRK2 kinase activity, leading to the hyperphosphorylation of RAB proteins, which are key regulators of intracellular trafficking and transport. Here, we review the current literature on how LRRK2 affects the axonal transport of different cargoes, focusing on synaptic vesicle precursors, mitochondria, and autophagosomes. We further discuss how LRRK2 influences cytoskeletal dynamics and how it affects vesicle trafficking at the Golgi, which may indirectly contribute to its effect on axonal transport. This review summarizes our current understanding of how pathogenic LRRK2 hyperactivation disrupts axonal transport and how this may be linked to the neurodegeneration of PD.

## Introduction

The human brain consists of billions of neurons, highly polarized cells that typically have several dendrites and one axon. The axonal arbor of a human CNS neuron is estimated to reach a total length of up to several hundred meters and to form up to several thousand synaptic connections with other neurons [1,2]. These dimensions of axonal length and connectivity make efficient transport mechanisms essential to ensure both the supply of material synthesized in the soma via anterograde transport and the removal of damaged organelles and misfolded proteins via retrograde transport.

Axonal transport relies on molecular motors, cytoplasmic dynein and kinesin, and on microtubules, the intracellular tracks used by these motor proteins. Kinesin motors belonging to kinesin-1, kinesin-2, kinesin-3, and kinesin-4 families have been implicated in mediating anterograde transport, while dynein controls retrograde transport of different cargo types, including organelles, synaptic vesicles, RNA, ribosomes, proteins, and lipids [3–5].

Disruptions in axonal transport have been linked to various neurodegenerative disorders, such as amyotrophic lateral sclerosis, Charcot–Marie–Tooth disease, Alzheimer’s disease, Huntington’s disease, and Parkinson’s disease (PD) [6–10]. PD is clinically characterized by the cardinal symptoms of tremor, rigidity, slowness of movements (bradykinesia), and postural instability [11]. While most PD cases are idiopathic, approximately 10–15% can be attributed to genetic mutations. A major genetic cause of familial PD is mutations in the leucine-rich repeat kinase 2 (*LRRK2*) gene located on chromosome 12 [12–14]. The encoded LRRK2 protein is a multidomain kinase involved in several intracellular processes, including the autophagy-lysosomal pathway, cytoskeletal dynamics, and vesicle trafficking [15].

LRRK2 consists of a GTPase domain (Ras of complex proteins [ROC] domain accompanied by a C-terminal of ROC [COR] domain) and a kinase domain, flanked by an armadillo repeat (ARM) region, an ankyrin repeat (ANK) region, a leucine-rich repeat (LRR) domain, and a WD40 domain. *LRRK2*-p.G2019S, the most common pathogenic mutation, is located within the LRRK2 kinase domain and induces LRRK2 kinase hyperactivity. There are six other very well-characterized PD-causing mutations, including four in the ROC domain (p.R1441C/G/H, p.N1437H), one in the COR domain (p.Y1699C), and one in the kinase domain (p.I2020T). Phosphorylation studies showed that all these mutations, which have been classified as ‘definitely pathogenic’, increase LRRK2 kinase activity [16]. The p.G2019S mutation increases kinase activity by about two-fold, whereas the other mutations increase it by three- to four-fold [17–20]. In addition to these seven well-characterized mutations, there are currently 45 other known mutations that increase kinase activity by at least 1.5-fold [21].

Hyperactive LRRK2 induces the hyperphosphorylation of a subset of RAB GTPases that have been characterized as *bona fide* substrates for LRRK2 [22,23]. RAB proteins play a crucial role in regulating intracellular transport, and an increasing number of studies link RAB hyperphosphorylation by hyperactive LRRK2 to defects in intracellular trafficking. Due to its length, the neuronal axon is predicted to be severely affected by any changes in trafficking and transport. In particular, dopaminergic neurons are characterized by highly branched axons, resulting in an extensive total length of their axonal arbor. This may make them particularly vulnerable to transport defects [24,25]. In this article, we review the current literature on how LRRK2 affects axonal transport and how disruption of axonal transport by pathogenic LRRK2 may contribute to the neurodegeneration underlying PD.

### LRRK2 and RAB GTPases

RAB GTPases are key regulators of intracellular vesicle trafficking and can exist in either an active (GTP-bound) or inactive (GDP-bound) form [26]. Prenylated, membrane-anchored RABs are activated by guanine nucleotide exchange factors, which induce conversion from the inactive GDP-bound state to the active GTP-bound state [27]. Upon activation, membrane- and GTP-bound RABs recruit downstream effector proteins to regulate various intracellular membrane-trafficking pathways [28]. RAB inactivation is catalyzed by GTPase-activating proteins, and inactive RABs are extracted from membranes by GDP dissociation inhibitors (GDIs) [27].

The transition from GTP-bound to GDP-bound state and vice versa is reflected by changes in the conformation of the RAB switch II domain, which plays an important role in mediating binding to effector proteins [29]. LRRK2 phosphorylates its RAB substrates within their switch II domain, thereby impairing their ability to interact with GDIs and their normal downstream effector proteins. Instead, LRRK2 phosphorylation enables RAB interaction with a new set of effector proteins that specifically bind to LRRK2-phosphorylated RAB proteins [22,23,30]. Effector proteins known to bind to LRRK2-phosphorylated RABs include RAB-interacting lysosomal protein (RILP)-like protein 1 (RILPL1), RILP-like protein 2 (RILPL2), c-Jun-amino-terminal kinase (JNK)-interacting protein 3 (JIP3) and JNK-interacting protein 4 (JIP4) [23,31,32]. Since only a small fraction of RAB GTPases are phosphorylated at endogenous LRRK2 expression levels, this dominant shift to a new set of effector proteins is currently considered to be the likely culprit for the effect of LRRK2 on intracellular trafficking pathways, rather than the phosphorylation-induced disruption of the interaction of RABs with their ‘normal’ effector proteins [21].

Ten RABs have been confirmed to be phosphorylated by LRRK2 under endogenous conditions: RAB3 (isoforms A-D), RAB8A/B, RAB10, RAB12, RAB35, and RAB43 [18,22,23,33]. In addition to their role as LRRK2 substrates, recent studies show that specific RABs also act upstream of LRRK2, recruiting it to membranes and subsequently activating its kinase activity. Membrane recruitment of LRRK2 via RAB interaction is mediated by binding of RABs to three distinct interaction sites, all located within the LRRK2 ARM domain. Site #1 binds (non-phosphorylated) RAB29/32/38 as well as RAB8 and RAB10 [34,35], whereas site #2 mediates interaction with phospho-RAB8 and phospho-RAB10 [35]. The recently discovered site #3 binds RAB12, mediating recruitment of LRRK2 to damaged lysosomes [36,37]. Together, these interactions support a feed-forward model, in which binding to non-phosphorylated RABs first mediates LRRK2 recruitment to membranes, where LRRK2 is then retained by interaction with phosphorylated RABs [21,35,38]. The pathological relevance of this upstream regulation of LRRK2 activity is illustrated by genetic evidence linking RAB29 and RAB32 to PD. The recently discovered *RAB32*-p.S71R mutation has been characterized as a familial PD risk factor and increases LRRK2 kinase activity when expressed *in vitro* [39,40]. RAB29 is located within the PARK16 locus, which is associated with familial PD [41,42].

In addition to the upstream regulation by RAB proteins, the lysosomal stress response termed ‘conjugation of ATG8 to single membranes’ (CASM) has recently been implicated in the recruitment of LRRK2 to damaged lysosomes [43,44]. Mechanistically, LRRK2 recruitment and activation at the lysosomal membrane has been linked to interaction with lipidated GABARAP, an ATG8 family member that is recruited to damaged lysosomes via CASM [43]. It is currently incompletely understood to what extent RAB- and GABARAP-mediated activation of LRRK2 acts interdependently or in parallel.

### LRRK2 and cytoskeletal dynamics

Microtubules are the intracellular tracks used by molecular motors to transport their cargo along the axon. They are dynamic structures formed by the polymerization of α- and β-tubulin heterodimers. A unique feature of axonal microtubules is their uniform arrangement, with their plus-ends (β-tubulin exposed) facing the axon terminal. First suggested by Gloeckner et al. [17], Kett et al. [45] were the first to show that overexpressed LRRK2 forms filamentous structures around microtubules [17,45]. The formation of these filaments is enhanced by overexpression of the *LRRK2* mutations p.N1437H, p.R1441G/C, p.Y1699C, and p.I2020T, but not by the p.G2019S mutation. Watanabe et al. [46] used cryo-electron tomography (cryo-ET) in combination with cryo-CLEM to analyze the structure of overexpressed LRRK2-p.I2020T bound to microtubules [46]. They found that LRRK2 forms a right-handed, double-stranded helix on left-handed microtubules. The association of LRRK2 with microtubules is mediated by interactions between basic residues of LRRK2’s ROC domain and the negatively charged microtubule surface [47].

Further work showed that the interaction between LRRK2 and microtubules is affected by the conformation of the LRRK2 kinase domain [48]. The kinase domain can be either open or closed, the latter promoting LRRK2’s oligomerization around microtubules. The LRRK2-specific type 1 kinase inhibitor MLi-2 stabilizes the closed conformation of the kinase domain and was found to promote the formation of microtubule-associated LRRK2 filaments in cells overexpressing WT LRRK2 [49–51]. In contrast, LRRK2 inhibitors that promote the open conformation (type 2 kinase inhibitors) are predicted to reduce the association of LRRK2 with microtubules [48].

To date, it is incompletely understood whether LRRK2 binding to axonal microtubules occurs at endogenous LRRK2 expression levels and, if so, how microtubule-associated endogenous LRRK2 affects axonal transport. Work in a cell-free *in vitro* system raised the possibility that MLi-2-induced formation of microtubule-associated LRRK2 patches may act as a roadblock that impairs the motility of molecular motors along microtubules [47]. However, studies in cultured primary and iPSC-induced neurons found that MLi-2 treatment did not impair axonal transport but rather rescued retrograde trafficking of autophagosomes in neurons expressing hyperactivating LRRK2 mutations or in neurons with knockout of the LRRK2 counteracting RAB phosphatase PPM1H [52–54]. While these results indicate that endogenous LRRK2 expression levels are too low to result in the formation of significant axonal transport roadblocks, it is currently unknown whether LRRK2 binding to microtubules may play other important roles in mediating the effect of LRRK2 on axonal trafficking, for example, by holding LRRK2 in proximity to substrates for activation. Exploring this possibility further and determining whether microtubule binding occurs at endogenous LRRK2 expression levels will be of great interest for future studies.

### Role of LRRK2 at the Golgi

The Golgi apparatus plays an important role in the sorting and packaging of vesicles destined for the axon. Several studies support a role for LRRK2 at the trans-Golgi network (TGN) in particular, where it may affect intracellular vesicle trafficking of the secretory pathway.

**Figure 1:**
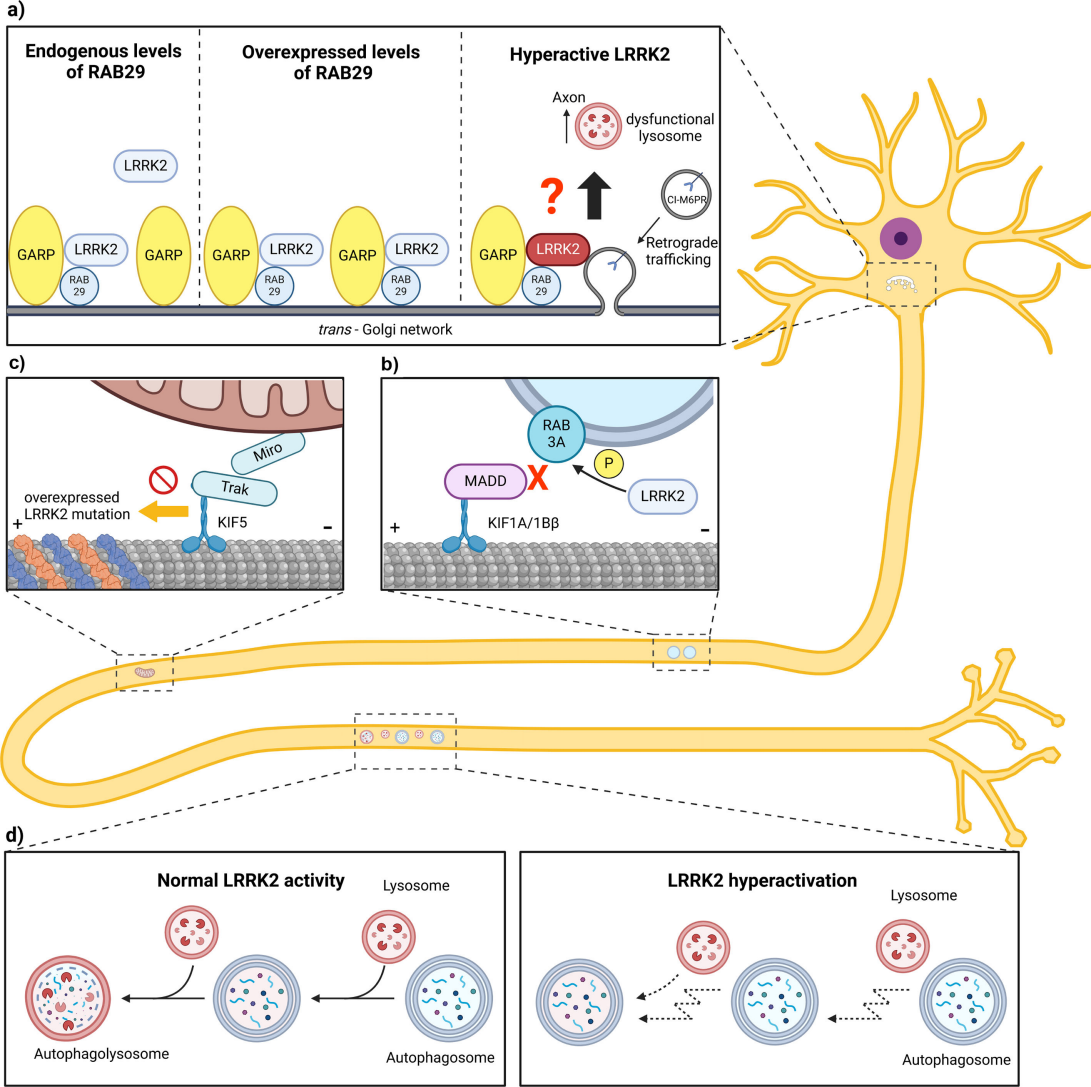
LRRK2’s effect on axonal transport. **(a**) LRRK2, recruited to the trans-Golgi network (TGN) by overexpressed RAB29, binds to the Golgi-associated retrograde protein (GARP) complex and affects the trafficking of the cation-independent mannose 6-phosphate receptor (CI-M6PR). Pathogenic hyperactivation of LRRK2 impairs sorting of lysosomal proteases at the Golgi, which may lead to the delivery of dysfunctional lysosomes to the axon. (**b**) Hyperphosphorylation of synaptic vesicle precursor (SVP)-associated RAB3A by hyperactive LRKK2 disrupts RAB3 binding to DENN/MADD and impairs the anterograde transport of SVPs. (**c**) Overexpressed LRRK2 mutations p.R1441C and p.Y1699C form filaments around deacetylated microtubules, blocking axonal transport of mitochondria. (**d**) Hyperactive LRRK2 disrupts the normally processive retrograde transport of autophagosomes, leading to impaired fusion with lysosomes and ultimately to insufficient degradation of autophagosomal cargo.

When overexpressed, the Golgi-resident RAB protein RAB29 recruits LRRK2 to the TGN and stimulates LRRK2 kinase activity [55–57]. At the TGN, LRRK2 has been found to bind to VPS52, a part of the Golgi-associated retrograde protein (GARP) complex. This interaction stabilizes binding of GARP to the t-SNARE STX-6 and affects retrograde and anterograde TGN transport in a LRRK2 kinase-dependent manner. Most notably, LRRK2 was found to affect trafficking of cation-independent mannose 6-phosphate receptor (CI-M6PR), which recognizes the M6P tag of newly synthesized lysosomal hydrolases and enables their sorting to endosomes for subsequent delivery to degradative lysosomes [58]. Analysis of brain tissue from PD patients with *LRRK2*-p.G2019S and *LRRK2*-p.I2020T mutations revealed reduced levels of CI-M6PR, suggesting that impaired sorting of lysosomal proteases at the Golgi may contribute to the impaired lysosomal function that has frequently been associated with LRRK2 PD [59,60]. It is currently unknown how the effect of LRRK2 at the Golgi affects vesicle trafficking in the axon. One possible scenario to explore further in future work is that defects in CI-M6PR trafficking at the TGN could result in a reduced degradative capacity of lysosomes entering the axon, possibly contributing to the impaired maturation of axonal autophagosomes reported previously [52,54] (Figure 1A).

An important limitation of studies on the role of LRRK2 at the Golgi is that robust LRRK2 recruitment to the Golgi and LRRK2 kinase activation have only been observed with strong transient overexpression of RAB29. The relevance of endogenous RAB29 for LRRK2 activation is currently unclear, as A549 cells with RAB29 KO showed only a modest reduction in LRRK2-phosphorylated RABs, and various tissues and primary cells from RAB29 KO mice showed no change in phospho-RAB levels [57,61]. Endogenous RAB29 is generally expressed at relatively low levels [21], and in the absence of RAB29 overexpression, only a small fraction of LRRK2 appears to localize to the Golgi [61]. Together with the other known RAB and non-RAB activators of LRRK2 (see ‘LRRK2 and RAB GTPases’), this may explain why RAB29 KO does not have a significant effect on whole-cell pRAB levels measured in cell or tissue lysates. In future work, it will be important to confirm whether endogenous RAB29 also mediates recruitment of LRRK2 to the Golgi and to investigate how the endogenous RAB29-LRRK2 axis affects vesicle sorting and trafficking at the Golgi.

Beyond its proposed functions at the Golgi, LRRK2 has also been functionally linked to the retromer, a multi-protein complex located on endosome membranes that regulates the recycling of endosomal transmembrane cargo to the TGN or plasma membrane. The PD-associated *VPS35*-p.D620N mutation, which affects the backbone VPS35 subunit of the retromer, has been shown by multiple groups to enhance LRRK2 kinase activity, although the underlying mechanism is incompletely understood [62–65]. Emerging evidence suggests that the p.D620N mutation, by impairing retromer-mediated retrograde trafficking, induces a specific form of lysosomal dysfunction and stress, which in turn may trigger LRRK2 membrane recruitment and kinase hyperactivation [66].

### LRRK2 and axonal transport of synaptic vesicle precursors

Synaptic vesicle precursors (SVPs) are transport vesicles that carry proteins and lipids essential for synaptic vesicle formation and neurotransmitter release [67]. These precursors are produced in the soma, transported along axons by molecular motors (kinesin-3 family members: KIF1A and KIF1Bβ), and develop into functional synaptic vesicles at presynaptic terminals [68,69]. It is estimated that each presynaptic site of a neuron is replenished with 20 to 40 new SVPs per day. A cholinergic neuron from the mouse CNS is estimated to have approximately 75,000 presynapses along its entire axonal arbor. This means that a total number of 1.5–3 million SVPs must travel a total distance of 2.8–5.6 km per day to replenish the presynapses of a single neuron [70]. These numbers are predicted to be even higher for dopaminergic neurons, which are estimated to have approximately 500,000 presynapses per neuron due to their extensive axonal branching and illustrate the need for highly efficient anterograde SVP transport [70].

Axonal transport of SVPs is dependent on RAB3, a GTPase that exists in the four isoforms RAB3A/B/C/D [69,71]. RAB3 forms a complex with the protein ‘differentially expressed in normal and neoplastic cells/MAP kinase activity death domain’ (DENN/MADD), which mediates the recruitment of kinesin motors to SVPs [72]. RAB3A-D isoforms are all directly phosphorylated by LRRK2 [22,23]. Studies in iPSC-derived human neurons found that hyperphosphorylation of RAB3 by the *LRRK2*-p.R1441H mutation reduces the anterograde transport of SVPs, leading to their accumulation in the soma and decreased delivery to presynaptic sites along the axon [73]. Knockout of the LRRK2-counteracting RAB phosphatase PPM1H had the same effect, implying a precise tuning of SVP transport by LRRK2 kinase and PPM1H phosphatase activity [73]. Mechanistically, the study found that phosphorylation of RAB3 disrupts its binding to DENN/MADD, which is predicted to prevent the formation of the RAB3–DENN/MADD–kinesin complex that normally drives anterograde SVP transport [73] (Figure 1B). These results are consistent with another recent study showing that pharmacological inhibition of LRRK2 kinase in WT neurons increases anterograde transport of alpha-synuclein, a presynaptic protein that aggregates in PD and is thought to play a physiological role in regulating neurotransmitter release [74].

In addition to its effect on axonal SVP transport, LRRK2 has been implicated in synaptic vesicles exocytosis and endocytosis at presynaptic sites [75] by interacting with several presynaptic proteins, including synapsin, voltage-gated calcium channels, NSF, endophilin-A, and its binding partner synaptojanin, as well as auxilin and clathrin adaptor protein 2 (AP2) [76–83]. Furthermore, the LRRK2-phosphorylated RAB proteins RAB3A [71,84–87], RAB5 [88–90], RAB10 [91,92], and RAB35 [93,94] have been found to be localized at the presynapse and implicated in presynaptic membrane trafficking. Overall, it is therefore likely that LRRK2 affects presynaptic function by altering both the axonal transport of SVPs and local membrane dynamics at the presynapse, possibly through both kinase-dependent and kinase-independent mechanisms. This is further complicated by the disruption of axonal autophagy by LRRK2-mediated RAB hyperphosphorylation (see ‘LRRK2 and axonal transport of autophagosomes’), which may affect the composition of presynaptic sites, as synaptic proteins have been identified as a major cargo of neuronal autophagosomes [95].

### LRRK2 and axonal transport of mitochondria

Mitochondrial transport is essential in axons, which require a constant supply of energy (ATP) and buffering of calcium. Axonal transport of mitochondria is dependent on motor proteins, kinesins (primarily kinesin-1) and dynein, which move mitochondria along the microtubule cytoskeleton [96–98]. The activity of these motor proteins is regulated by a set of motor adaptor proteins. A complex of the proteins TRAK and Miro links mitochondria to kinesin-1 and dynein, while Syntaphilin acts as an anchor to stop mitochondria at specific locations [99,100]. Mitochondria do not move continuously but rather adapt their movement to local energy demands and calcium levels, leading to their accumulation in metabolically demanding regions such as synapses and growth cones [101,102].

Axonal trafficking is in need of a significant amount of energy in the form of ATP to power molecular motors. LRRK2 is a component of the ER at axonal mitochondria-associated membranes (MAMs), which promotes Ca2+ transfer and lipid exchange, making it crucial for mitochondrial bioenergetics [103,104]. LRRK2 normally blocks the degradation of MAM components by binding the E3 ubiquitin ligase. However, when LRRK2 kinase activity is increased, as is the case with the p.G2019S mutation, E3 ubiquitin ligase is released, and proteasomal degradation of MAM components is promoted. In addition, mitochondrial energy production is down-regulated by a decrease in ER-mitochondrial Ca2+ transfer due to loss of ER-mitochondria contact [103].

Several studies have reported that pathogenic mutations in *LRRK2* affect the axonal transport of mitochondria. Upon overexpression of LRRK2-p.R1441C or LRRK2-p.Y1699C, but not LRRK2-p.G2019S, Godena et al. observed decreased motility of axonal mitochondria in rat primary neurons and Drosophila motor neurons [105]. This transport impairment was associated with the formation of LRRK2 filaments around deacetylated microtubules (Figure 1C). Treatment with deacetylase inhibitors prevented the formation of LRRK2 filaments and rescued axonal transport [105]. Mechanistically, it seems plausible that the impairment of mitochondrial transport observed in this study represents a non-specific disruption of axonal transport by the formation of LRRK2 filaments around microtubules, rather than a specific effect on mitochondrial transport. The finding that overexpression of LRRK2-p.R1441C and LRRK2-p.Y1699C, but not LRRK2-p.G2019S, caused transport impairment is consistent with this idea, since the p.R1441C and p.Y1699C mutations, but not the p.G2019S mutation, are known to induce filament formation of overexpressed LRRK2 [45]. As discussed in ‘LRRK2 in cytoskeletal dynamics’, the formation of LRRK2 filaments around microtubules has so far only been observed upon overexpression of LRRK2.

At endogenous expression levels, a recent study found no effect of the expression of hyperactive LRRK2 on the axonal transport of mitochondria in both human iPSC-derived neurons and mouse primary neurons. Neither *LRRK2*-p.G2019S KI nor *LRRK2*-p.R1441H KI significantly affected mitochondrial transport in the axon of human iPSC-derived iNeurons [53]. Moreover, no differences were observed when comparing axonal transport of mitochondria in mouse primary WT and *LRRK2*-p.G2019S KI neurons, and when assessing the effect of the LRRK2 kinase inhibitor MLi-2 on mitochondrial transport in primary WT and p.G2019S KI neurons [53].

Hsieh and colleagues also found no difference in mitochondrial motility under baseline conditions but observed an impaired arrest of axonal mitochondria following mitochondrial damage by antimycin A treatment in iPSC-derived dopaminergic neurons from *LRRK2*-p.G2019S patients [106]. Mechanistically, the group proposed that WT LRRK2 induces the removal of Miro, an adaptor protein that connects mitochondria to molecular motors, from the outer mitochondrial membrane upon mitochondrial damage. The p.G2019S mutation disrupts the interaction of LRRK2 with Miro, delaying mitochondrial arrest and thereby slowing the subsequent degradation by mitophagy. This effect was not rescued by pharmacological inhibition of LRRK2 kinase activity [106]. A more recent study assessing mitophagy in the soma observed impaired basal mitophagy and delayed degradation of Miro in *LRRK2*-p.R1441C but not *LRRK2*-p.G2019S neurons [107].

In sum, these data suggest that LRRK2 may play a more important role in regulating mitochondrial transport under conditions of mitochondrial stress than under baseline conditions, but more studies are needed to decipher the impact of different mutations in LRRK2 at increasing levels of mitochondrial damage in detail. Since there is currently no known involvement of LRRK2-phosphorylated RABs in regulating mitochondrial motility, it seems likely that any effects of LRRK2 on mitochondrial transport are either mediated through RAB-independent mechanisms or secondary to other effects of hyperactive LRRK2.

### LRRK2 and axonal transport of autophagosomes

Macroautophagy (autophagy) is a process by which misfolded proteins, damaged organelles, and other unwanted cellular components are degraded and recycled in a cell type-specific manner [95]. Autophagy is essential for neuronal health as it mediates the degradation of intracellular debris, providing new building blocks for future components and acting as a quality control mechanism that removes damaged organelles. Autophagy begins with autophagosome biogenesis, the formation of a double-membrane vesicle that engulfs autophagosomal cargo. Subsequently, autophagosomes fuse with lysosomes, delivering their cargo for degradation by lysosomal proteases.

Autophagy in neurons is characterized by several unique features. Neuronal autophagosomes are primarily formed in the distal axon and at presynaptic sites, where autophagosome biogenesis occurs at higher rates compared with the mid-axon and the neuronal soma [108–111]. Axonal autophagosomes form constitutively under fed conditions, and their formation does not show a robust up-regulation in response to nutrient starvation. This is in stark contrast to the induction of autophagy upon starvation in many non-neuronal cell types [112,113].

Once formed, neuronal autophagosomes undergo processive retrograde axonal transport toward the soma, driven by the retrograde motor dynein. Both the retrograde motor dynein and the anterograde motor kinesin are bound to the autophagosome membrane, but a set of motor adaptor proteins promotes dynein activity while inhibiting kinesin activity to enable processive autophagosome transport in the retrograde direction [108,109,114,115]. In contrast to the robust retrograde motility of autophagosomes, axonal lysosomes display more heterogeneous dynamics: approximately half are stationary, while the mobile fraction is evenly split between anterograde and retrograde movement [116,117]. During retrograde autophagosome transport, autophagosomes fuse with lysosomes, which lowers the intra-lumenal pH and activates degradative enzymes [115,118,119] (Figure 1D). Processive retrograde axonal transport is tightly linked to efficient autophagosome maturation, as disruption of autophagosome transport by knockdown of key motor adaptor proteins is associated with impaired autophagosome acidification and impaired degradation of autophagosomal cargo [120,121].

Since early work observed an accumulation of autophagosomes in cells expressing pathogenic LRRK2 mutations [122–124], several studies have proposed a role of LRRK2 in the autophagy–lysosome pathway [125–134]. In previous work of our group, we found that hyperactive LRRK2 disrupts the normally processive retrograde axonal transport of autophagosomes, impairing autophagosome maturation and the degradation of autophagosomal cargo [52–54] (Figure 1D).

We observed in both primary neurons and human iPSC-derived iNeurons that expression of LRRK2-p.G2019S disrupts retrograde autophagosome transport by increasing the number of pauses and directional reversals during retrograde autophagosome motility [52]. iNeurons with *LRRK2*-p.R1441H KI, a mutation known to induce even higher levels of kinase hyperactivity than p.G2019S, showed a similar but more severe transport deficit, suggesting that higher levels of LRRK2 kinase activity lead to increased disruption of axonal autophagosome transport [53]. Loss of the LRRK2-counteracting RAB phosphatase PPM1H phenocopied the effect of hyperactive LRRK2 in both primary neurons from a PPM1H KO mouse model and in iNeurons derived from gene-edited PPM1H KO iPSCs. In both model systems, defective autophagosome transport in PPM1H KO neurons was rescued by the LRRK2-specific kinase inhibitor MLi-2 [53,54]. Together, these findings strongly indicate that the disruption of autophagosome transport by hyperactive LRRK2 is mediated by hyperphosphorylation of RAB proteins. Of note, primary neurons with homozygous loss of PPM1H showed a more pronounced deficit than neurons with heterozygous loss of PPM1H, further supporting the idea that higher levels of LRRK2 kinase activity and RAB hyperphosphorylation scale with more severe disruption of autophagosome transport [54].

Downstream of RAB hyperphosphorylation, the dysregulation of autophagosome transport has been attributed to an imbalance between JIP4, a motor adaptor protein that specifically binds to LRRK2-phosphorylated RAB proteins [32,52,135], and the small GTPase ARF6. JIP4 recruits and activates the anterograde motor kinesin, unless binding to ARF6 blocks JIP4’s interaction with kinesin and instead favors activation of the retrograde dynein/dynactin complex [136]. LRRK2-mediated RAB hyperphosphorylation induces an increased recruitment of JIP4 to the autophagosomal membrane, which recruits and activates kinesin in the absence of ARF6 regulation, inducing an unproductive ‘tug-of-war’ between anterograde and retrograde motor proteins attached to the autophagosome membrane that disrupts processive retrograde organelle motility [52,53]. This model is supported by the finding that transient overexpression of ARF6 attenuates the defect in autophagosome transport in both neurons expressing hyperactive LRRK2 and in neurons with loss of PPM1H [53]. Conversely, ARF6 knockdown in WT neurons reduces retrograde autophagosome motility, consistent with a key role of ARF6 in the regulation of autophagosome transport [115].

In contrast to its effect on autophagosome transport, the p.G2019S mutation was not found to affect the axonal transport of LAMP1-positive lysosomal vesicles [52]. Notably, the motility of LAMP1 vesicles in WT neurons is overall less processive than that of autophagosomes; approximately 50% of axonal LAMP1 vesicles are stationary or move bidirectionally, and 25% are transported in both the anterograde and retrograde direction, whereas 80% of autophagosomes move processively in the retrograde direction [116,117,137]. These baseline differences in transport dynamics may explain why RAB hyperphosphorylation, JIP4 recruitment, and downstream signaling steps—though potentially occurring at both organelles—only manifest as a measurable transport defect in autophagosomes. In future work, it will be interesting to investigate whether more hyperactivating LRRK2 mutations such as *LRRK2*-p.R1441H, which causes a more pronounced disruption of autophagosome transport than p.G2019S, also induce a significant defect in LAMP1 vesicle motility. Supporting a potentially broader role of RAB proteins that are phosphorylated by LRRK2, a recent study found that overexpression of RAB10 in WT neurons affects the axonal transport of both autophagosomes and LAMP1 vesicles [115].

How might the disruption of autophagosome transport by hyperactive LRRK2 be linked to the neurodegeneration underlying PD? Consistent with previous work showing that processive retrograde transport of autophagosomes is tightly linked to effective autophagosome maturation [120,121,138], disruption of autophagosome transport by hyperactive LRRK2 was associated with impaired autophagosome acidification, resulting in a reduced fraction of acidified autophagosomes specifically in the proximal axon [52]. Notably, this defect may become increasingly important with age, as the rate of autophagosome formation has been shown to significantly decrease in neurons from aged mice [139,140]. Furthermore, a recent study suggests that the impairment of autophagosomal degradation by pathogenic LRRK2 may lead to a compensatory up-regulation of secretory autophagy, resulting in increased extracellular release of autophagic cargo. This increased secretion of intracellular waste may have short-term benefits for neuronal health, but in the longer term may contribute to disease progression and spread of pathology [141].

An autophagosomal cargo of particular interest is alpha-synuclein (aSyn), a key protein in PD pathophysiology and established marker of disease progression [142–144]. Primary neurons from *LRRK2*-p.G2019S KI mice exhibit elevated levels of detergent-insoluble endogenous aSyn, along with increased extracellular release of aSyn into the culture medium [145]. Moreover, several studies demonstrated that neurons expressing hyperactive LRRK2 show increased formation of aSyn aggregates upon treatment with aSyn preformed fibrils (PFFs) to seed aggregate formation [146–150]. Our recent work found that impaired autophagosome transport caused by loss of the LRRK2-counteracting RAB phosphatase PPM1H was accompanied by defective autophagosomal degradation of axonal aSyn and exacerbated aSyn aggregation upon treatment with PFFs, an effect that was dependent on LRRK2 kinase activity [54]. These findings link LRRK2-mediated RAB hyperphosphorylation to aSyn pathology in PD and suggest that impairment of axonal autophagy may contribute to the increased vulnerability of neurons expressing pathogenic LRRK2 to aSyn aggregation. An interesting open question is whether LRRK2’s effect on autophagosomal degradation and the compensatory up-regulation of secretory autophagy in the soma and primary dendrite [141] are also tied to tau pathology, which has been found to show enhanced retrograde spread along neuroanatomical connections in brains of *LRRK2*-p.G2019S mice and is more prevalent than aSyn pathology in LRRK2 PD cases [151–153].

### Perspectives/Conclusions

Several studies have reported that axonal damage and synaptic dysfunction in the striatum precede the loss of cell bodies in the substantia nigra pars compacta (SNc) in PD, supporting a ‘dying back’ disease model of retrograde axonal degeneration [6,154–158]. This pattern is consistent with the idea that the disruption of axonal transport induced by pathogenic LRRK2 contributes to PD pathogenesis. Interestingly, alterations in axonal transport and other intracellular processes occur even though only a small fraction of RAB GTPases is phosphorylated—even in cells expressing hyperactive LRRK2 [18]. These observations may be partly explained by the emerging concept that LRRK2-mediated phosphorylation shifts RAB interactions to a new set of effector proteins, thereby exerting dominant effects on intracellular trafficking pathways [21]. Further elucidating how phospho-RABs, despite low phosphorylation stoichiometry, drive broader effects on intracellular trafficking will be an important objective for future studies.

In addition to their effect on axonal transport, LRRK2 mutations have been associated with altered organelle dynamics in the soma, such as the response to lysosomal membrane damage, ciliogenesis/centriole cohesion, and mitophagy. Emerging data show that other central nervous system cell types, particularly glial cells, also play an important role in PD pathogenesis [135,159–164]. In future work, it will be of great interest to investigate to what extent the effect of LRRK2 on these different intracellular pathways is cell type-specific and what role non-cell autonomous effects play in the pathophysiology of PD.

## Open Access

This article has been published open access under our Subscribe to Open programme, made possible through the support of our subscribing institutions, learn more here: https://portlandpress.com/pages/open_access_options_and_prices#conditional

## Abbreviations

COR: C-terminal of ROC
LRRK2: leucine-rich repeat kinase 2
PD: Parkinson’s disease
ROC: Ras of complex proteins
